# Dispersion of SARS-CoV-2 lineage BA.5.1.25 and its descendants in Peru during two COVID-19 waves in 2022

**DOI:** 10.1186/s44342-024-00006-3

**Published:** 2024-05-31

**Authors:** Victor Jimenez-Vasquez, Natalia Vargas-Herrera, Luis Bárcena-Flores, Verónica Hurtado, Carlos Padilla-Rojas, Roger V. Araujo-Castillo

**Affiliations:** Centro Nacional de Salud Pública, Instituto Nacional de Salud, Capac Yupanqui 1400-Jesus Maria, Lima, Peru

**Keywords:** Genomics, Surveillance, COVID-19, SARS-CoV-2, Omicron, Peru

## Abstract

During the third year of the pandemic in Peru, the persistent transmission of SARS-CoV-2 led to the appearance of more transmissible and immune-evasive Omicron sublineages; in that context, the National Genomic Surveillance of SARS-CoV-2 performed by the Peruvian National Institute of Health detected spike mutations in the circulating Omicron BA.5.1.25 sublineage which was later designated as DJ.1 and increased during the fourth COVID-19 wave, this eventually branched into new sublineages. The introduction, emergence, and timing of the most recent common ancestor (tMRCA) of BA.5.1.25 and its descendants (DJ.1, DJ.1.1, DJ.1.2, and DJ.1.3) were investigated in this paper as well as the time lags between their emergence and identification by the Peruvian National Institute of Health. Our findings show that ongoing genomic surveillance of SARS-CoV-2 is critical for understanding its phylogenetic evolution and the emergence of novel variations.

## Introduction

Peru has experienced an unprecedented number of cases and one of the highest mortality and lethality rates worldwide due to COVID-19 [1]. The number of cases and deaths have been clustered in five epidemic waves until the end of 2022; the first two showed high hospitalization and mortality rates and were associated with the initial SARS-CoV-2 lineages and a native variant Lambda (C.37) [2]. The subsequent three waves were linked to the Omicron variant (B.1.1.529), exhibiting less clinical severity; while the third wave was caused by the Omicron parental and BA.1 lineages; the last two presented a high percentage of Omicron BA.5 lineages, which have been the most prevalent around the globe until January 2023 according to epidemiological reports [3, 4].

These lineages have been distinguished from other Omicron sub-variants due to the Δ69-70, L452R, F486V, and R493Q spike mutations, which confer higher transmissibility and further immune evasion from the responses elicited by vaccination and previous BA.1 and BA.2 infections [5–7]. Subsequently, BA.5 continued evolving into new sub-lineages, some of them different enough to be monitored independently by the World Health Organization (WHO) and the Centers for Disease Control and Prevention (CDC) due to their threat to global public health [8]. These included BQ.1 and BQ.1.1, which caused a surge of cases in Europe and America in October 2022 and raised concern because of their capability to evade the immune system [9].

In Peru, the continuous transmission of SARS-CoV-2 led to the accumulation of new mutations and, as result, the emergence of 24 native lineages, including the Lambda variant. This phenomenon compelled the Peruvian Genomic Surveillance working group to wonder if the last two epidemic waves (fourth and fifth) were associated with the worldwide circulating BA.5 lineages, to the emergence of new mutations, or the appearance of new BA.5 clades. The ensuing phylogenetic analysis allowed us to identify the emergence of the epidemiological behavior of a rather unremarkable lineage into a concerning subvariant.

## Methods

### Epidemiological assessment

Since 2021, the Peruvian National Institute of Health (INS) has accumulated information about the SARS-CoV-2 genomic surveillance which is been presented in the weekly updates [10], which also include trends in the SARS-CoV-2 variants. This information was gathered from weekly samples of 350 sequenced nasopharyngeal swabs taken randomly from ambulatory and hospitalized patients across the country (80% from Lima and 20% from other regions). Additionally, the SARS-CoV-2 genomic sequencing from the INS is distributed across four sites in Peru: the INS Biomedicine laboratory in Lima, the National Reference laboratories in the central highlands of Junin and Cusco, and the Mobile Laboratory in the country’s northwestern city of Piura.

### Sequencing and phylogenetic reconstruction

As part of the genomic surveillance of SARS-CoV-2 in Peru, nationally weekly compiled nasopharyngeal swab samples obtained from June to December of 2022 were processed using the Illumina COVIDSeq kit and sequenced using the NextSeq 550 and MiniSeq platforms. The resulting fastq files were assembled by mapping with the reference Wuhan-2 (GenBank accession: NC_045512.2) with the programs BWA v0.7.17 [11], samtools v1.13 [12], and iVar v1.3.1 [13]. We estimated sequencing depth and genomic coverage with samtools v1.13 and seqtk v1.3. A total of 1065 genomes were identified as belonging to the BA.5.1.25 or its descendants DJ.1, DJ.1.2, DJ.1.3, and DJ.1.1. We additionally downloaded 4453 genomes identified as BA.5.1.25 or BA.5.1 from the GISAID database (https://gisaid.org/) [14]. A total of 5524 genomes were processed with nextclade in order to identify nucleotide mutations, amino acid mutations, percentage of missing data, and genomic coverage. We obtained a global phylogenetic tree with RAxML v8.2.12 [15] by running 20 independent searches; this tree was finally inspected in microreact (microreact.org).

### Phylochronology

Temporal signal (clocklikeness) of the data was assessed with TempEst v.1.5.3 [16] with the following procedure: a total of 50 genetic and time representative tips of the clade of interest were visually identified in the global phylogeny; the respective genomes were individualized and subjected to a phylogenetic reconstruction with the maximum likelihood (ML) algorithm available in RAxML v8.2.12 with 20 independent runs. Sampling dates were transformed to decimal format with R package; “lubridate” and clocklikeness were tested by inspecting the correlation coefficient (root to tip divergence versus time) under the best-fitting root algorithm. A time calibrated Bayesian analysis was conducted under the skyline coalescent model, a strict molecular clock model, and the HKY + I + G substitution model using two independent Markov chain Monte Carlo (MCMC) runs consisted of 50 million generations with a sampling of 5000 generations each and default prior parameters, in BEAST v1.10.4. [17, 18]. Effective sampling sizes (ESS) over 200 were inspected in TRACER v1.7.1 [19]. A burning of 10% was used, and the remaining data was merged in Log-Combiner v1.10. A summary time-calibrated tree was obtained with TreeAnnotator v.1.10 [17]; the resulting tree was visualized and edited in FigTree v1.4.

## Results

Regarding epidemiological information from these Omicron sublineages, in total, 1029 samples sequenced in Peru until December 2022 resulted in either BA.5.1.25, DJ.1, DJ.1.1, DJ.1.2, or DJ.1.3 and were collected mostly in Lima (41.97%), Loreto (31.55%), Ucayali (8.83%), and Ancash (5.35%) regions. Additionally, 56.65% of the samples were collected from female patients and 43.35% from male patients, with a median age of 42 years and an interquartile range of 29 and 54. Regarding vaccination status, 95.30% (*n* = 974) had been vaccinated with only one dose, 94.32% (*n* = 964) received only two COVID-19 vaccine doses, 89.24% (*n* = 912) received three COVID-19 vaccine doses, 49.02% (*n* = 501) received four vaccine doses, and only 12.82% (*n* = 131) received five COVID-19 vaccine doses. In these patients, two 70- and 65-year-old females from Loreto vaccinated with three doses died from COVID-19.

**Fig. 1:**
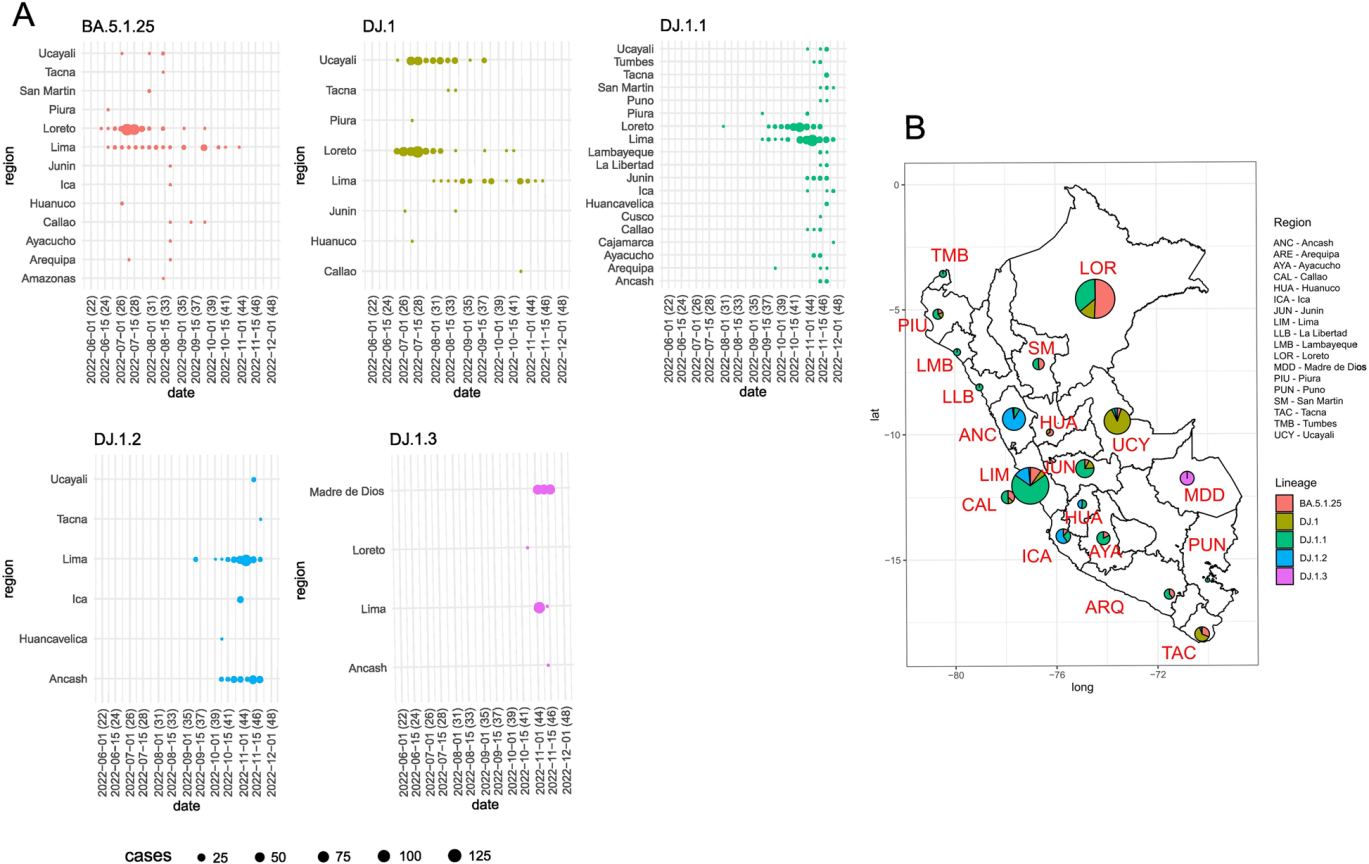
Epidemiological week findings

Our findings presented in Fig. 1 show that in epidemiological week (EW) 24, which corresponds to the second week of June 2022, the first records of the BA.5.1.25 lineage were detected in Loreto, Lima, and Piura regions, and by the end of June (EW 25), the first genomes carrying the spike mutation K444N were detected in the Loreto and Ucayali Amazonian regions. This clade was subsequently designated as DJ.1. In the beginning of August (EW 31), the first genomes containing the spike mutation N460K (T22942G and C21757T) were discovered in Loreto and within DJ.1. As a result, this new lineage was given the name DJ.1.1 and was extended to 19 regions throughout the country until the end of November 2022 (EW 48). Additionally, a novel clade harboring the spike mutations N460K (T22942G) and L176K (C14599T and C22088T) was found in Lima (EW 36) and Ancash (EW 40) regions, with limited circulation in Huancavelica, Ica, Ucayali, and Tacna regions until the middle of November (EW 46): this was called DJ.1.2. Finally, by the end of October (EW 42), a third clade harboring ORF1ab mutation T3287I (C10125T) and spike mutation N460K (A22942G) was designated DJ.1.3 and was connected to a genome circulating in Lima City and predominantly detected in Madre de Dios region.

**Fig. 2:**
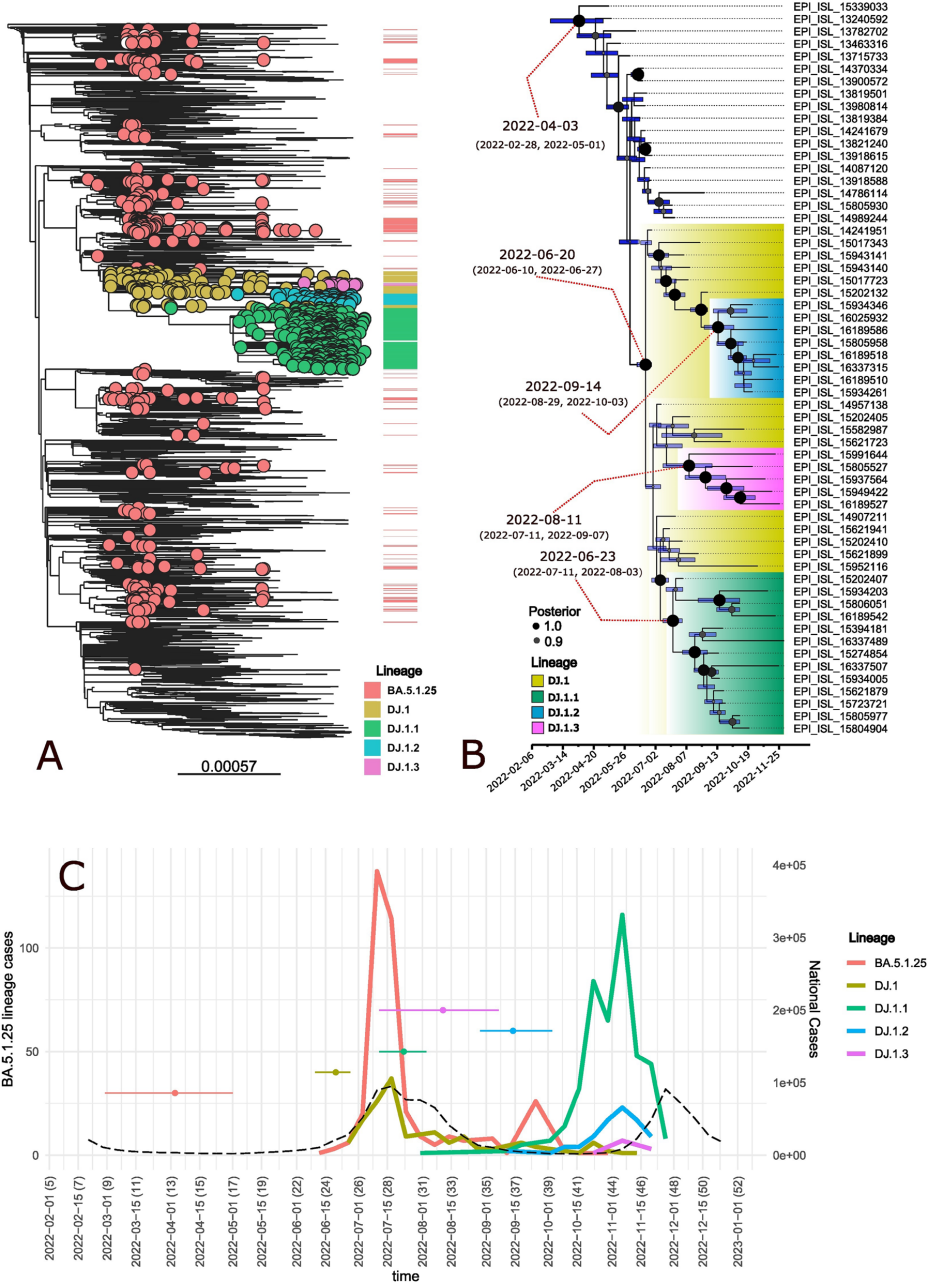
Global maximum likelihood (ML) phylogenetic reconstruction

According to the global maximum likelihood (ML) phylogenetic reconstruction depicted in Fig. 2A, Peru faced several independent introductions (red dots); the approximately six remaining BA.5.1.25 introductions resulted in minor cases, the majority of which lasted until September and a few until November. One introduction was responsible for the formation of the DJ.1 lineage and descendants, and our analysis recognizes sample EPI_ISL_14087120 (a patient from Utah, USA, sampled on June 26) as the potential ancestor.

The maximum clade credibility tree (MCCT) depicts the Bayesian phylochronology analysis, which estimates the genesis of the BA.5.1.25 lineage by April 3 (95% high probability density (HPD): February 28–May 1) (Fig. 2B). DJ.1 was estimated to have emerged on June 20 (95% HPD: June 10–June 27), lineage DJ.1.1 was projected to have emerged on July 23 (95% HPD: July 11–August 3), DJ.1.2 was estimated to have emerged on September 14 (95% HPD: August 29–October 3), and DJ.1.3 was estimated to have emerged on August 11 (95% HPD: July 11–September 7). This information together with actual dates of detection and dispersion over time are presented in Fig. 2C and Table 1.

**Table 1:** Estimated Bayesian origin dates and 95% probability densities (upper and lower) of the BA.5.1.25 lineage and descendants

Lineage	Oldest detected sample (EPI code)	Lower 95% HPD	Median	Upper 95% HPD
BA.5.1.25	May 5, 2022 (EPI_ISL_15339033)	February 28, 2022	April 3, 2022	May 1, 2022
DJ.1	June 28, 2022 (EPI_ISL_14241951)	June 10, 2022	June 20, 2022	June 27, 2022
DJ.1.1	August 4, 2022 (EPI_ISL_15202407)	July 11, 2022	July 23, 2022	August 3, 2022
DJ.1.2	September 14, 2022 (EPI_ISL_15274852)	August 29, 2022	September 14, 2022	October 3, 2022
DJ.1.3	October 20, 2022 (EPI_ISL_15729372)	July 11, 2022	August 11, 2022	September 7, 2022

## Discussion

In Peru, the continuous transmission of SARS-CoV-2 has led to the accumulation of new mutations and, as a result, a significant phylogenetic evolution of certain Omicron sub-lineages, such as BA.5.1.25. This descendant lineage of BA.5 was first identified in our country during EW 24 of 2022, predominantly in the Loreto region, but was also present in Lima and Ucayali regions. BA.5.1.25 presented a steep increase between the EW 26 and 27 (corresponding to early to July of 2022 and the fourth COVID-19 epidemic wave in Peru). When generic COVID-19 cases increased throughout the country as the fifth wave manifested, DJ.1 descendant lineages DJ.1.1, DJ.1.2, and DJ.1.3 dropped by the end of October 2022 (EW 44–45). Phylogenetic analysis performed by the Peruvian genomic surveillance group found that the spike mutation N460K was presented in the lineage DJ.1.2 by the end of October; this mutation has been found in BQ.1 and BQ.1 sublineages causing neutralization resistance and increased spike affinity [18]; the majority of cases of this lineage was registered in Lima and Ancash regions. Subsequently, the designated DJ.1.1, DJ.1.2 (this last one had the additional mutations N:G5E and L176K), and DJ.1.3 sublineages were associated with the rise in COVID-19 cases observed in Loreto. The DJ.1 lineage would have appeared for the first time in EW 26–2022 and then started to rise in EW 26 to 28–2022 along with BA.5.1.25 to reach a peak in EW 28–2022. Subsequently, DJ.1 started to decrease and was last detected in EW 6–2023.

The association between the fourth COVID-19 wave, the entrance of the BA.5.1.25 lineage in Peru, and the formation of the DJ.1 lineage is clearly depicted in Fig. 2C. Curiously, our study shows that the DJ.1 lineage emerged roughly a week before the initial case was reported, and the BA.5.1.25 lineage emerged just over a month before it was found in Peru. The estimations of the origins of the DJ.1.2 and DJ.1.3 lineages present the greatest deviations even though the origin of the first coincides with the first record for this lineage, while for the second, we found a difference of almost 70 days between the origin and the first record. This contrasts with DJ.1.1, whose estimate of origin differs from the first record by less than 10 days (Table 1). This disparity could be explained by a smaller number of DJ.1.2 and DJ.1.3 cases in comparison with DJ.1.1. As has been observed since the beginning of the COVID-19 pandemic, tMRCA estimations have been conducted by ML [20] or Bayesian approaches [21], not to estimate the date of origin of SARS-CoV-2 but to address the late emergence of variants and lineages [22], and our estimations present similar deviations found in those studies, ranging from weeks to less than few months.

It is possible that weak preventive measures such as the non-use of facemasks, the lack of social distance, and low vaccination coverage contributed to the increase in BA.5.1.25 lineage cases in the Loreto region between June and July 2022. Curiously, the “optional use of masks” was implemented on May 1, 2022, 1 month after the end of the third wave (EW 13) in Peru, as a precaution for those who had received the third COVID-19 vaccine dose. However, by that time, the Loreto region registered the following vaccination levels: 84.06% for one dose, 75.21% for two doses, 39.6% for three doses, and 0.82% for four doses, compared with the Ancash region, which registered 95.88%, 92.85%, 67.05%, and 1.30%, respectively, according to the COVID-19 vaccination board from the National Repository of Health Information (REUNIS) [23].

We consider that inconsistent use of facemasks and low third COVID-19 vaccine coverage may have contributed in the spread of the Omicron lineages and the subsequent appearance of its descendants, who took part in the fourth and fifth waves as well. The decrease in BA.5.1.25 cases and its descendants during EW 30–2022 to 37–2022 depicted in Fig. 1C may be attributed to fewer samples received for genomic sequencing during that time. This highlights the significance of bias-free genomic surveillance sampling in order to produce a graph that accurately depicts the actual transmission patterns of the lineages.

The BA.5.1.25, DJ.1, and DJ.1.1 lineages occurred as parallel but independent events in time with a predominance in Loreto first and then in Lima, which have a geographical distance of 1012 km; their main communication channel is by airplane. In the case of the DJ.1 lineage, several cases also occurred in Ucayali, which is 676 km from Loreto, connected by airplane and river. The DJ.1.2 and DJ.1.3 lineages were predominant first in Lima, then in Ancash (433.2 km) and Madre de Dios (678 km) respectively.

Our results reinforce the importance of continuing genomic surveillance in order to track the SARS-CoV-2 genomic evolution in a given territory. As shown in this paper, not only worldwide recognized variants can be associated to an increment of cases but also domestic lineages with a favorable mutation profile can contribute to local but large epidemic waves. Therefore, genomic surveillance should not be limited to identify and follow well-described variants; it should also carefully evaluate any lineage that shows unusual behavior. This assessment should focus in locating any consistent deviations from the expected nucleotide profile and search for known high-risk mutations. If a locally relevant lineage is recognized, enhancing public health measures is essential to reduce the transmission of the virus, especially vaccination. In the long-term, these measures ought to stop mutation accumulation and perpetuation of an ever-changing SARS-CoV-2 virus.
